# HBOS-CNV: A New Approach to Detect Copy Number Variations From Next-Generation Sequencing Data

**DOI:** 10.3389/fgene.2021.642473

**Published:** 2021-06-07

**Authors:** Yang Guo, Shuzhen Wang, Xiguo Yuan

**Affiliations:** The School of Computer Science and Technology, Xidian University, Xi’an, China

**Keywords:** copy number variations, next-generation sequencing data, outlier detection, histogram analysis, tumor purity

## Abstract

Copy number variation (CNV) is a genomic mutation that plays an important role in tumor evolution and tumor genesis. Accurate detection of CNVs from next-generation sequencing (NGS) data is still a challenging task due to artifacts such as uneven mapped reads and unbalanced amplitudes of gains and losses. This study proposes a new approach called HBOS-CNV to detect CNVs from NGS data. The central point of HBOS-CNV is that it uses a new statistic, the histogram-based outlier score (HBOS), to evaluate the fluctuation of genome bins to determine those of changed copy numbers. In comparison with existing statistics in the evaluation of CNVs, HBOS is a non-linearly transformed value from the observed read depth (RD) value of each genome bin, having the potential ability to relieve the effects resulted from the above artifacts. In the calculation of HBOS values, a dynamic width histogram is utilized to depict the density of bins on the genome being analyzed, which can reduce the effects of noises partially contributed by mapping and sequencing errors. The evaluation of genome bins using such a new statistic can lead to less extremely significant CNVs having a high probability of detection. We evaluated this method using a large number of simulation datasets and compared it with four existing methods (CNVnator, CNV-IFTV, CNV-LOF, and iCopyDav). The results demonstrated that our proposed method outperforms the others in terms of sensitivity, precision, and F1-measure. Furthermore, we applied the proposed method to a set of real sequencing samples from the 1000 Genomes Project and determined a number of CNVs with biological meanings. Thus, the proposed method can be regarded as a routine approach in the field of genome mutation analysis for cancer samples.

## Introduction

Copy number variation (CNV) is a type of structural variation in human genomes that accounts for a large part of the genome diversity and is associated with many complex human diseases (Feuk et al., 2006), such as autism, Parkinson’s disease, schizophrenia, and cancer. CNVs are generally defined as amplifications or deletions in DNA fragments larger than 1 Kb and can span up to 1 Mbp, accounting for 12–16% of the entire human genome (Redon et al., 2006). Traditionally, CNVs were identified with cytogenetic technologies such as karyotyping and fluorescence *in situ* hybridization (FISH), array comparative genomic hybridization, or single nucleotide polymorphism array approaches (Itsara et al., 2009). However, these methods are sub-optimal because of hybridization noise, limited genome coverage, and low resolution. Different from traditional methods, the short reads generated by next-generation sequencing (NGS) technologies have a higher resolution that provides potential advantages for the accurate detection of CNV regions as small as several hundred bases (Metzker, 2010; Zhao et al., 2013).

In recent years, numerous classic methods for detecting CNV from NGS data have been developed, such as FREEC proposed by Boeva et al. (2012), which uses GC-content to normalize read counts from tumor samples, and automatically determines a window size for each sample. FREEC can estimate the tumor purity of the sequenced samples and predict the genotype for each genomic segment. However, because the automatically defined window size might be very volatile, the breakpoint positions of the detected CNVs might be different from reality (Boeva et al., 2012). One of the most popular read depth (RD)-based methods is CNVnator (Abyzov et al., 2011). CNVnator uses the mean-shift (Comaniciu and Meer, 2002) method to cluster RD data and segments the signals after clustering. After that, adjacent segments are merged with minimal difference in the average RD by a greedy algorithm (Abyzov et al., 2011). Finally, CNVs are called via a *t*-test procedure. The algorithm has the advantage of high precision and fast speed. However, when there is relatively low coverage depth data, the false positive rate of CNVnator is difficult to control due to the influence from artifacts such as an uneven distribution of reads and a difference in bandwidth size. The iCopyDav method developed by Prashanthi et al. (2018) divides the genome into small intervals according to the RD signal and defines the upper and lower thresholds to determine gain or loss. It possesses good sensitivity and precision but requires high sequence coverage and high tumor purity.

Different from the above methods, CNV-IFTV (Yuan et al., 2021) proposed by Yuan et al. (2019) is a CNV detection algorithm based on isolated forest and total variation models. The algorithm uses the prior knowledge that the CNV region is far smaller than the normal region to accurately detect the CNV region. First, each bin is graded by the isolated forest, and then, the continuous bins are smoothed by the total variation model. Based on the first step, the significant distribution value is calculated to call the CNV (Yuan et al., 2021). However, many factors related to CNV, such as the inherent correlations among genome positions, have not yet been fully explored by the method. Besides, due to the insufficient utilization of computing performance, the algorithm runs for a considerable length of time. Another method using multi-threading is CNV-LOF (Yuan et al., 2019), this method adds positional information to the processed RD signals to convert them into two-dimensional data and uses the local outlier factor (LOF) algorithm to determine the local outliers. This method explores the relationship between copy number amplitude and positional space, and it possesses good precision and sensitivity for low tumor purity data and exhibits low time complexity. However, the sensitivity of the algorithm is too high, which leads to additional false-positive results for high tumor purity data.

Although these methods provide significant results, none of them is sufficiently versatile in various scenarios, such as when there is (1) spatial dependence among consecutive bins that can weaken the difference of the RD value between normal and abnormal regions. Many existing methods ignore the correlation and interaction among continuous bins and do not detect CNVs with a small fluctuation of RD values. (2) Due to the difference in the amplitudes between gains and losses, the distribution of the RD profile is not well-fitted by statistical models (Miller et al., 2011). Many methods use linear or non-linear transformations to process RD profiles to better fit statistical models. However, the unbalanced signals still affect the processed profile, resulting in a high false discovery rate. (3) When the coverage depth or tumor purity is low, most methods cannot maintain stable detection results. Many methods produce satisfactory detection results using sequencing data with high coverage depth and high tumor purity, but few CNVs have been detected in low tumor purity and low coverage data (Telenti et al., 2016). Another drawback of the methods is that although they improve the detection precision of low-quality data, the false-positive rate is difficult to control when there are high purity and high coverage data (Chen et al., 2017).

With careful consideration of the issues mentioned above, in this study, we propose a new approach to detect copy number variations, called Histogram-based Outlier Score of Copy Number Variation (HBOS-CNV). HBOS-CNV uses a histogram to analyze the RD value of each bin in the whole genome. Unlike the standard histogram with equal intervals, the interval for each column in the histogram of HBOS-CNV is dynamic (Goldstein and Dengel, 2014). Thus, HBOS-CNV can reduce the influence from unbalanced signals and calculate the density of each bin. Besides, to ensure the inherent correlations among genome positions of the impression. Before using a histogram, the discrete 1D convolution kernel is used to smooth the bins in sequence data. This method highlights the difference in RD value between normal and abnormal bins, and also satisfactorily reflects the relationship between copy number amplitude and positional space.

To demonstrate the power of our method, we used HBOS-CNV to analyze simulation datasets and real WGS datasets and compare their performance to that of four other state-of-the-art tools. All performed analyses demonstrated that our approach is capable of detecting CNV in low coverage depth and low tumor purity datasets, thus outperforming all other compared tools.

## Materials and Methods

### Flowchart of the HBOS-CNV Method

**FIGURE 1:**
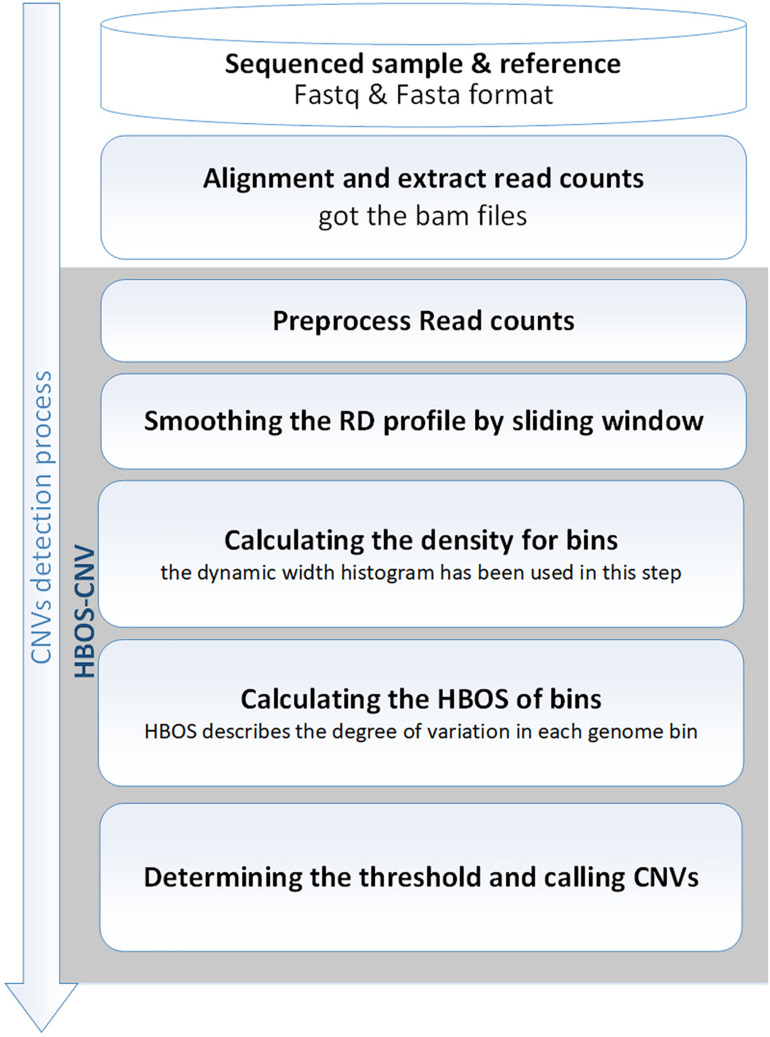
Diagram showing the HBOS-CNV workflow. It includes the main steps for processing input read count profiles from SAMtools: preprocessing, smoothing, calculating the density of bins, calculating the HBOS, determining the threshold, and calling the CNV.

HBOS-CNV is based on RD and performs the analysis of CNV without the requirement of control-matched samples. Before running HBOS-CNV, it is necessary to use the BWA software (Li and Durbin, 2010) to align a sequencing sample with a reference genome (such as hg19). Then, the read count profile is extracted using the SAMtool software (Li et al., 2009), and stored in a BAM file. Based on the BAM file, HBOS-CNV detects CNV through the following five processes. Figure 1 shows an overview of the HBOS-CNV process, as follows. (1) *The read counts profile is preprocessed*. In this step, we address the lost values and the ‘N’ position in the gene sequence, generating bins and RD signals, and correcting the GC content bias. (2) *The RD profile is smoothed by a sliding window*. By smoothing the RD value, the noise in the RD contour is reduced, and the interaction in continuous bins is considered. (3) *The density of bins is calculated*. The dynamic width histogram is used in this step to compute the density of bins. (4) *The HBOS of bins is calculated*. The HBOS is used to evaluate the degree of outliers. (5) *The threshold is determined, and CNVs are declared*. According to the HBOS profile, the threshold of the normal interval is determined, and the CNVs are called with the threshold. HBOS-CNV software is implemented in Python, and the code can be found at https://github.com/BDanalysis/HBOS-CNV. In the following subsections, the implementation of the proposed method is described in detail, and then, the characteristics of the approach are discussed.

### Preprocessing

For the read count profile obtained from a BAM format file, preprocessing is initially required. Most preprocessing methods are similar and mainly divided into four steps. In the first step, the ‘N’ positions are removed from the reference genome (Backenroth et al., 2014; Wang et al., 2014). In the second step, the read count profile is divided into non-overlapping bins with the same length (Backenroth et al., 2014). In the third step, the mean read count is calculated as a read depth (RD) value in each bin. In the last step, the GC bias correction is conducted via Eq. 1 (Yoon et al., 2009; Abyzov et al., 2011):

(1) $$r_{m}=\frac{\bar{r}}{{\bar{r}}_{g\,c}}\cdot {\tilde{r}}_{m},$$

where *r*_*m*_ represents the corrected RD values of the *m*-th bin and ${\tilde{r}}_{m}$ represents the raw values of the *m*-th bin. The average of the RD values across all bins is denoted by $\bar{r}$ is meant; ${\bar{r}}_{g\,c}$ represents the average RD value of those bins with similar GC fractions to the *m*-th bin. In this study, we used a previously described method for the GC bias calculations (Yu et al., 2016; Poell et al., 2018; Yuan et al., 2018a).

### Smoothing the RD Profile by Sliding Window

After preprocessing, in order to ensure that the following process does not produce new errors, we need to divide the gaps caused by telomeres and centromeres from the preprocessed data (Montpetit et al., 2014). Firstly the RD frequency in the RD profile is clustered into two clusters with K-means (Hartigan and Wong, 1979). Secondly, the preprocessed data is roughly divided into two segments according to the average RD value represented by these clusters. Then, the discrete 1D convolution kernel is used to smooth each segmentation. Generally, the RD value of the *m*-th bin has the greatest influence on itself, and the influence of the other neighboring bins weakens in turn, which is similar to the normal distribution. Therefore, we design the weight of the convolution kernel to be a normal distribution. Finally, all segments are stitched together in the initial order to form a complete smoothed sequence. The calculation for the sliding window is shown in Eq. 3. Each recalculated bin, named $r_{m}^{\prime }$, represents the new statistic for the *m*-th bin in the RD profile:

(2) $$\begin{array}{c} \vec{R}=\left(r_{m-w},r_{m-w+1},\ldots ,r_{m+w}\right)\\ \vec{X}=\left(x_{m-w},x_{m-w+1},\ldots ,x_{m+w}\right),\vec{X}∼N\,\left(\mu ,\sigma \right),\\ r_{m}^{\prime }=\vec{R}\cdot \vec{X} \end{array}$$

where $r_{m}^{\prime }$ represents the statistic of the *m-*th bin, which is obtained by the inner product of two vectors. $\vec{\text{R}}$ represents the RD value of the bins in the sliding window, *w* represents half the length of the sliding window, and $\vec{\text{X}}$ represents the weight of normal distribution. *N*(μ,σ) represents a normal distribution with the expectation of μ and a standard deviation of σ. Generally, the value of μ is 0, which can ensure that the RD value of a bin has the greatest impact on itself. The size of *w* is set to 0.01% of the total number of bins. In other words, the size of *w* depends on the size of bin-size. Since the chromosome length is fixed, the smaller the bin-size, the larger the value of *w*. The value of the standard deviation σ depends on the Eq. 3:

(3) $$\sigma =\frac{1}{\bar{r}}\cdot \frac{\sum_{i=1}^{n}\left|r_{i}-\bar{r}\right|}{n},$$

where *n* represents the number of total bins in the chromosome, $\bar{r}$ represents the average of RD, *r*_*i*_ denotes the RD value of *i-*th bin.

### Calculating the Density of Bins

For the RD profile obtained after sliding window processing, a dynamic width histogram is used to describe the bin density on the analyzed genome, which can ensure that the density calculation is based on a local fixed number of bins. The result is minimally affected by the unbalanced signal in the global RD values. The specific algorithm is as follows. It is necessary to set the number of columns in the histogram, and we can ensure the number of bins in each column via Eq. 4:

(4) $$S=\frac{n}{k},$$

where *n* denotes the number of total bins in the chromosome, *S* represents the number of bins in each column, and *k* denotes the number of columns (Goldstein and Dengel, 2014). Generally, the number of columns *k* is set as the square root of the number of total bins *n* (Goldstein and Dengel, 2014).

The first step is sorting the bins according to the RD value, and arranging the *S-*sorted consecutive bins into a single column. Because the area of a column in a histogram represents the number of bins, it is the same for all columns in the histogram (Goldstein and Dengel, 2014). At the same time, the width of the column is defined by the first and the last value in the column. Thus, the height of each column can be computed via Eq. 5 (Goldstein and Dengel, 2014). This indicates that columns covering a larger interval of the RD value exhibit decreased height and represent a lower density:

(5) $$h\,i\,s\,t\,\left(i\right)=\frac{S}{\left(r_{\max}^{i}-r_{\min}^{i}\right)+1},$$

where *hist*(*i*) represents the height of the *i*-th column in the histogram, *S* represents the number of bins in each column, and $r_{m\,a\,x}^{i}$ and $r_{m\,i\,n}^{i}$ represent the max and min read depths, respectively, in the *i*-th column. Add one to the denominator to make sure the denominator is not zero. Finally, the histograms are then normalized such that the maximum height is 1.0. This ensures an equal weight of each feature concerning the outlier score (Goldstein and Dengel, 2014).

However, there is an exception. Under the influence of centromeres and telomeres, there are usually no mapped reads at some positions of chromosomes. In this case, the RD values of these bins are all 0, and the number of these bins is more than S. Therefore, the method allows having more than *S* values in the same column (Goldstein and Dengel, 2014). Significantly, in this case, the calculated *hist(i)* value is very large, so the HBOS value (used to measure outliers) calculated by *hist(i)* will be very small. In CNV detection, this method can avoid the gap caused by telomere and centromere to be detected as CNV.

### Calculating the HBOS of Bins

Finally, according to the height of each column, the outlier factor of bins in the column is calculated via Eq. 6. That is, the outlier of each bin is determined by the outlier of the column in which it is located:

(6) $$H\,B\,O\,S\,\left(i\right)=\log\left(\frac{1}{h\,i\,s\,t\,\left(i\right)}\right)$$

where *HBOS*(*i*) represents the score of the *i-*th column in the histogram, and *hist*(*i*) represents the height of the *i*-th column.

**FIGURE 2:**
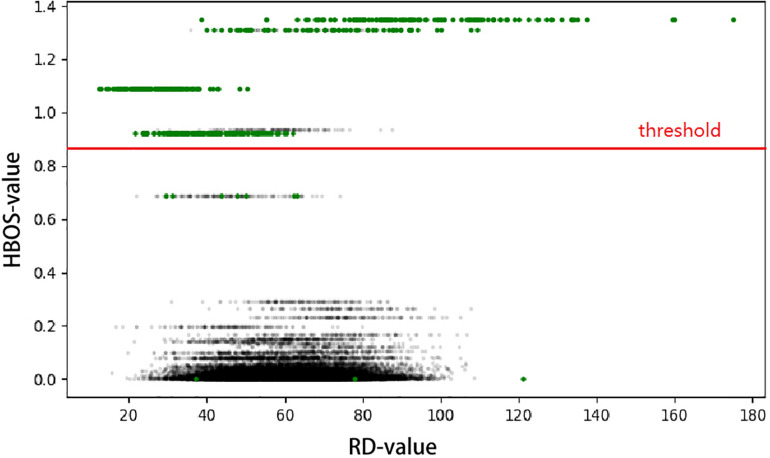
The RD value was used as the *x*-axis and the HBOS of each bin as the *y*-axis to construct a scatter plot. The generated profile can greatly separate the normal area and the variation area. The green point is the ground truth of CNV, and the parts above the red line are predicted as CNV regions.

This result, called the Histogram-Based Outlier Score (HBOS), is a non-linearly transformed value from the observed RD value of each genome bin. HBOS describes the degree of variation in each genome bin. With an increasing possibility of mutation in genomic bins, HBOS will become larger. As shown in Figure 2, the green points indicate the ground truth of CNVs, and the black points are the normal bins. When we use RD signal (*x*-axis) to directly detect CNV, half of the CNVs are covered in the normal area, which will be detected by HBOS signal (*y*-axis).

### Determining the Threshold and Calling CNVs

The HBOS is not a binary property, and therefore, it cannot be used to directly determine CNVs. It is necessary to design a stable and reliable method to declare CNVs based on the HBOS profile. The commonly used method, which assumes a null distribution and calculates the *p*-value for each object, determines outliers by a significance level. However, this method requires that the data be subject to the corresponding distribution model (Chen and Yuan, 2020). The distribution of the HBOS profile is complex, and it cannot be subject to the null distribution model. To overcome this limitation, the method used to calculate the upper limit in the boxplot is used to determine the threshold of outliers in the HBOS profile (Yuan et al., 2019). A value greater than the threshold in the HBOS profile is judged as the outlier. Finally, outliers are mapped to the RD profile to determine the position of CNVs.

After the declaration of CNVs, the gain or loss in the variation region needs to be determined. It is similar to the algorithm we previously developed (Yuan et al., 2021). In this study, the mean RD value is used as the criterion to decide the gain or loss. The mean RD value ($\bar{r}$) is calculated over all bins by removing the variation bins. The method of calculating the absolute copy number is shown in Eq. 7:

(7) $$C\,N=\frac{\left(r_{t}-\left(1-\varphi \right)\cdot \bar{r}\right)\cdot \rho }{\varphi \cdot \bar{r}},$$

Where the ρ represents the tumor ploidy, in the data of the human genome, it is usually assumed to be 2, *r*_*t*_ represents the average RD of the CNV region, and the φ represents the tumor purity. Similar to most algorithms (Cun et al., 2018), the tumor purity can calculate by the RD of hemizygous loss (*r*_*hem*_), homozygous loss (*r*_*hom*_), and average RD ($\bar{r}$). The method is shown in Equation 8, the tumor purity φ is the average of ‘φ_*1*_andφ_*2*_:

(8) $$r_{\text{hem}}=\varphi_{1}\cdot \frac{\bar{r}}{2}+\left(1-\varphi_{1}\right)\cdot \bar{r},r_{\mathrm{hom}}=\left(1-\varphi_{2}\right)\cdot \bar{r}$$

## Results

### Simulation Study

#### Simulation Datasets

Simulation studies are considered to be an appropriate method for evaluating the performance of existing and new methods (Yuan et al., 2012, 2018b). To evaluate the rationality and reliability of this method, a unified evaluation criteria should be adopted, and the comparison method should have the same input. With that in mind, we compared HBOS-CNV with four existing methods (CNVnator, CNV-IFTV, CNV-LOF, and iCopyDav) to obtain sensitivity, precision, and F1-measure (harmonic mean of the sensitivity and precision). The simulation software named IntSIM (Yuan et al., 2017), a simulation tool we previously developed was used to generate various datasets with tumor purity ranging from 0.2 to 0.6 and sequencing coverage ranging from 4 to 8 times. In each simulation configuration, 50 duplicate samples were generated to fully test the five methods. In each replicate sample, 14 CNVs were simulated, ranging in size from 10,000 to 500,000 BP. Besides, all experiments in this chapter use hg18 as the human reference genome.

#### Parameters of the Methods

To ensure a comprehensive comparison, we set the bin-size of 500 and 1000 when we used simulation data to test these methods. Specifically, in addition to setting the bin-size, other parameters are set as follows: (1) HBOS-CNV uses the square root of the number of total bins as the default *k* value, which is defined as the number of columns. (2) In CNVnator, the four parameters (*-his*, *-stat*, *-partition*, and *-call*) are the same as bin-size in the experiment. (3) The parameter of CNV-IFTV to control the number of isolated trees is 256 by default. (4) The default value of *segCount* defined by LOF-CNV is 50, and the default value of k is 10. (5) In iCopyDav, the *minSize* is consistent with the bin-size in the experiment, and the *genome flag* is set to *hg18*.

#### Simulation Experiments and Comparison With Peer Methods

**FIGURE 3:**
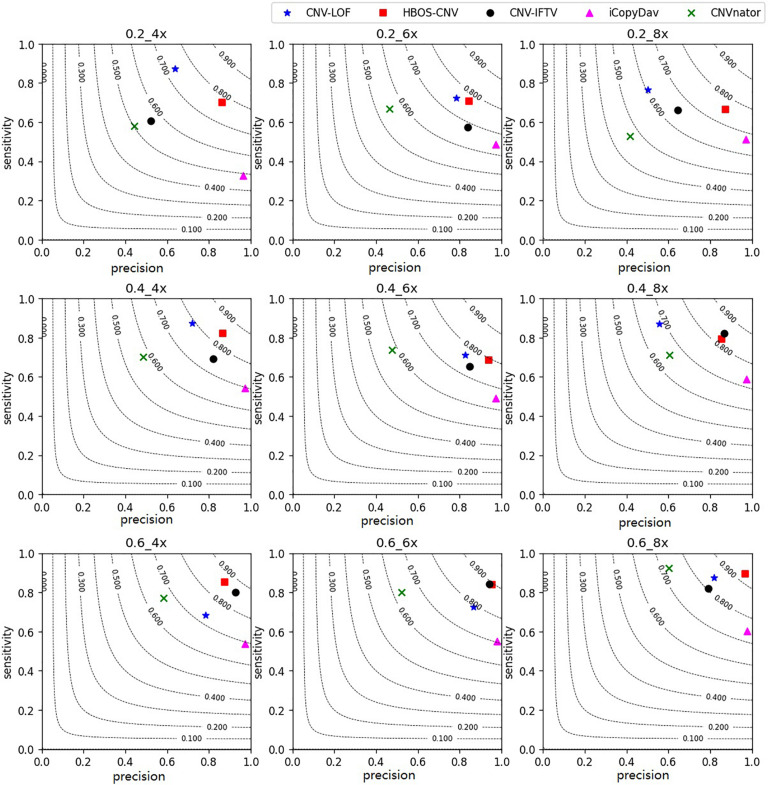
Performance comparisons between the five methods with bin-size of 1000 in terms of sensitivity, precision, and F1-measure on simulation datasets. The F1-measure levels are shown in grey curves ranging from 0.1 to 0.9.

**FIGURE 4:**
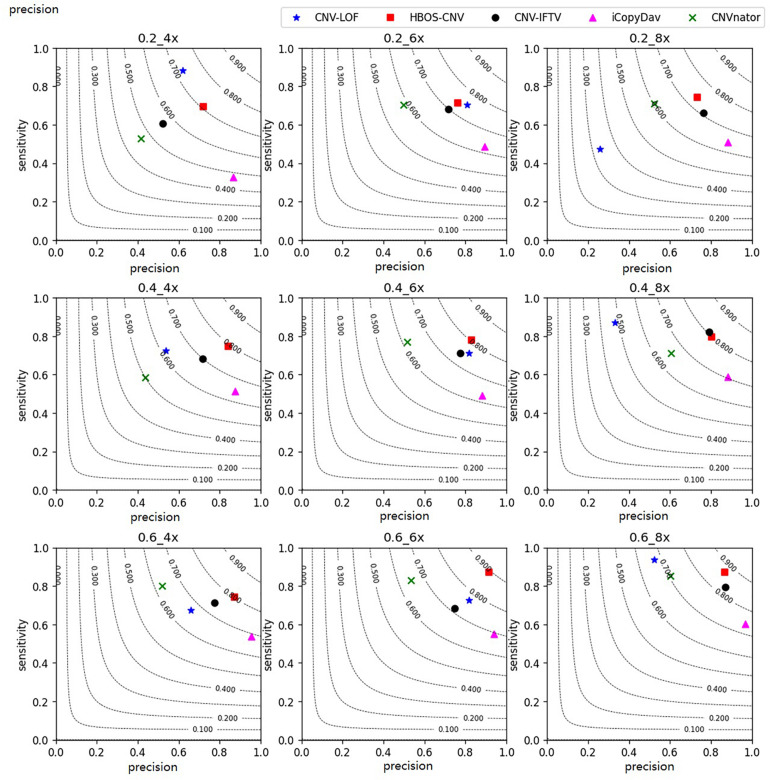
Performance comparisons between the five methods with the bin-size of 500 in terms of sensitivity, precision, and F1-measure on simulation datasets. The F1-measure levels are shown in grey curves ranging from 0.1 to 0.9.

With the simulation datasets, we performed all five methods described herein. They revealed the sensitivity, precision, and F1-measure, which were the average of the 50 repeated samples running results. The program running results for the simulated data are shown in Figures 3, 4. Figure 3 shows the precision and sensitivity of the detection results of all methods when the bin-size is 1000. When the bin-size is 500, the detection results are shown in Figure 4. The tumor purity of the data was 0.2, 0.4, and 0.6. The simulated data coverage depth was 4 times, 6 times, and 8 times respectively.

In the figures, for the data with low tumor purity and low coverage, the results are quite different. For these five methods, with the improvement of tumor purity, the change of CNV-LOF was very small or even decreased, the precision and sensitivity of CNV-IFTV were greatly improved, CNV-LOF only maintained a high sensitivity, and iCopydav only maintained a high precision. Different from the other four methods, the HBOS-CNV method not only ensured the precision and sensitivity of high-purity data, but there was also a reliable result in low-purity data.

As shown in Figure 3, for the low coverage data, such as 4 times, the F1-measure value of HBOS-CNV was the largest, and CNV-LOF was the second largest when the tumor purity was low. However, when the tumor purity was high, the F1-measure value of HBOS-CNV was the largest, and CNV-IFTV was the second largest. The F1-measure value of CNV-LOF underwent little change. Figure 4 shows the results of the five algorithms when the bin-size is 500. The bin-size has a certain impact on the results of HBOS-CNV, which is mainly reflected in a 10% decrease in precision. However, considering the precision and sensitivity, the F1 value of HBOS-CNV is still the first. In terms of precision, the result of HBOS-CNV was the second most precise one of the algorithms. The most precise algorithm was iCopydav, and the other algorithms produced results that were lower in precision than those of HBOS-CNV. In terms of sensitivity, HBOS-CNV was second only to CNV-LOF in the case of low purity. Considering only precision or sensitivity, HBOS-CNV may not be the optimal solution, but considering both, the F1-score of HBOS-CNV is the first. In other words, HBOS-CNV has high precision and sensitivity in low coverage and low purity sequencing samples.

For the high coverage data, such as 8 times, all the five algorithms have high sensitivity and precision. When the bin-size is 1000, The precision and sensitivity of HBOS-CNV rank first or second, especially in the data of high tumor purity, HBOS-CNV has the best results, and its F1-score is close to 0.93. Comparing Figures 3, 4, the result shows that when the bin-size changes, the F1-value of the HBOS-CNV method is at a relatively stable level. In the high coverage data, changing the bin-size reduces the precision by about 5% at most, while the precision reduces close to 10% in the low coverage data. When the sensitivity and precision of the simulation results were considered, HBOS-CNV exhibited the most optimal trade-off in the detection of various purity levels.

**FIGURE 5:**
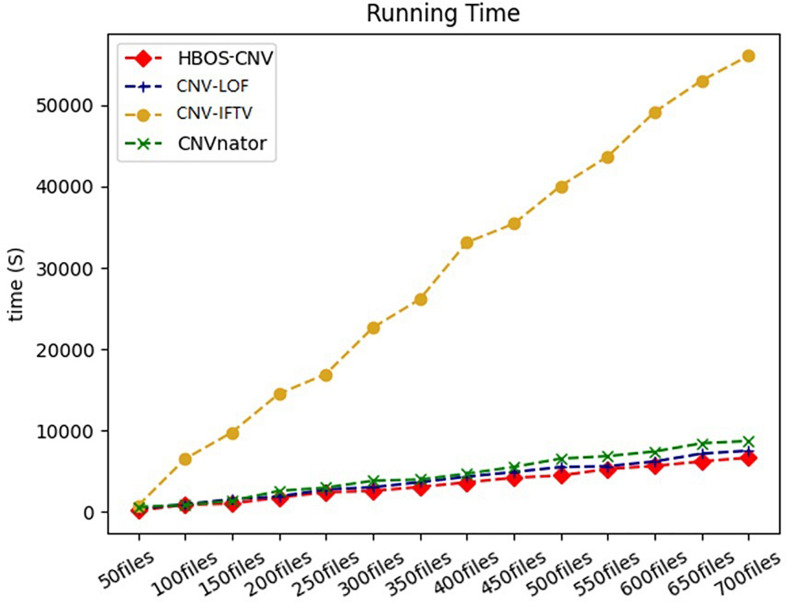
The running time of the five methods was compared by running 50 to 700 files. Each file records the simulated sequencing data of chromosome 21, and the size of each file is about 1G.

In addition to the above three criteria, the running time of the algorithm was compared to measure the detection efficiency. We prepared 100 to 700 copies of simulation data. Under the condition of ensuring the accuracy of the results, we ran the above five algorithms and recorded their running times. The results are shown in Figure 5, and illustrate that CNV-IFTV has the longest running time. CNV-IFTV makes insufficient use of computer computing performance, and thus, the running time is high, and the time significantly increases with the increase in files. Other algorithms, such as CNVnator, CNV-LOF, iCopyDav, and HBOS-CNV, exhibit low complexity and relatively high efficiency. Combined with precision, sensitivity, and running time, HBOS-CNV is the optimal compromise.

### Real Data Applications

#### The Analysis of Samples From the 1000 Genomes Project

To examine the effectiveness of HBOS-CNV, it was applied to analyze real sequencing samples that were obtained from the 1000 Genomes Project^1^ (Handsaker et al., 2015; Eberle et al., 2017). We selected three representative detection results by analyzing the samples of father, mother, and child from the same family, to show the comparison results between the proposed method and the other four methods (CNVnator, iCopyDav, CNV-LOF, and CNV-IFTV). According to the CNVs of these chromosomes reported in the DGV database (MacDonald et al., 2014)^2^, we calculated the sensitivities, precisions, and F1-measures for the five compared methods so that we could accurately evaluate these five methods.

**FIGURE 6:**
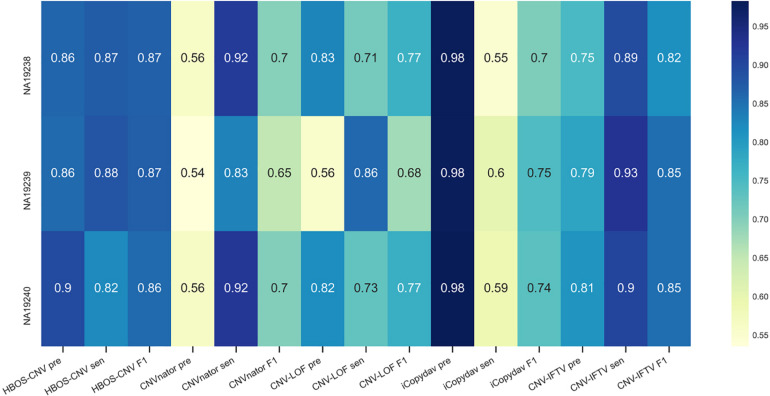
The *x*-axis represents the precision, sensitivity, and F1-score of the five methods, and the *y*-axis represents the three samples (NA19238, NA19239, NA19240). The precision, sensitivity, and F1-score of each method are marked in the figure.

The precision, sensitivity, and F1 value of each sample are shown in Figure 6. Among the three samples, considering precision and sensitivity, HBOS-CNV has the highest F1-score, and CNV-IFTV ranks second. Considering only sensitivity, CNVnator maintains the highest value, and only considering accuracy, iCopydav has the highest value. Therefore, HBOS-CNV exists as the optimal trade-off in real data.

**FIGURE 7:**
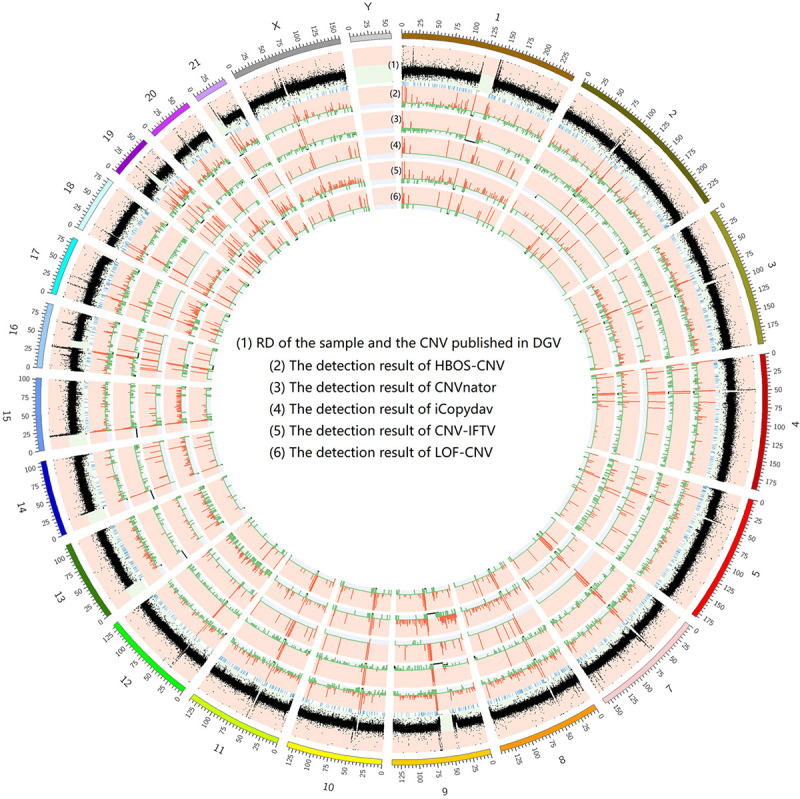
The detection of all whole genomes of NA19238 is shown, with the circles representing (1) RD, and CNV published in DGV (2) the CNVs detected with HBOS-CNV, (3) the CNVs detected with CNVnator, (4) the CNVs detected with iCopyDav, and (5) the CNVs detected with CNV-IFTV, (6) the CNVs detected with LOF-CNV, from the outside to the inside.

**FIGURE 8:**
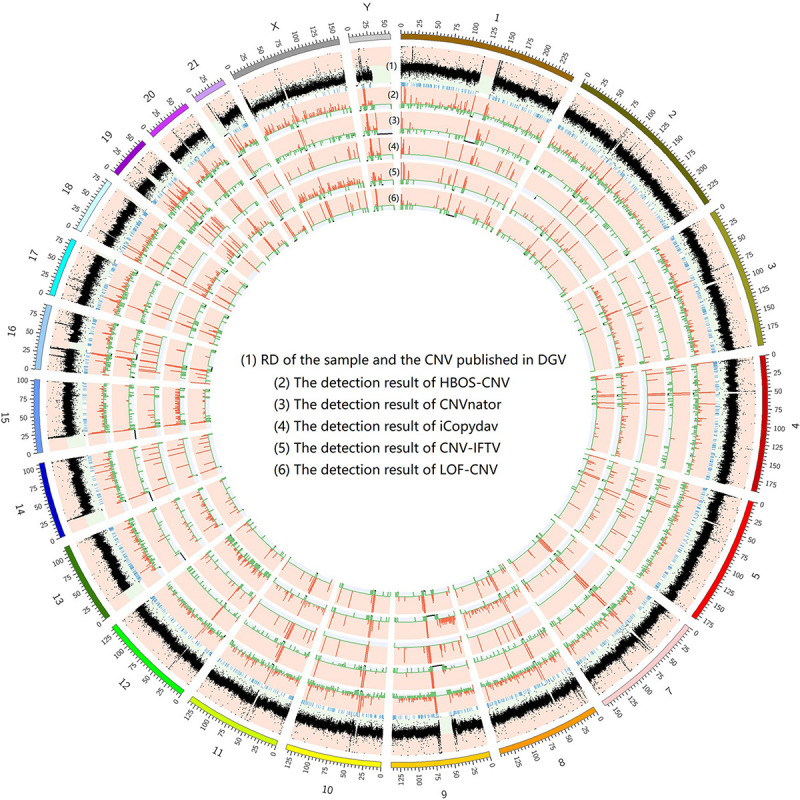
The detection of all whole genomes of NA19239 is shown, with the circles representing (1) RD, and CNV published in DGV (2) the CNVs detected with HBOS-CNV, (3) the CNVs detected with CNVnator, (4) the CNVs detected with iCopyDav, and (5) the CNVs detected with CNV-IFTV, (6) the CNVs detected with LOF-CNV, from the outside to the inside.

**FIGURE 9:**
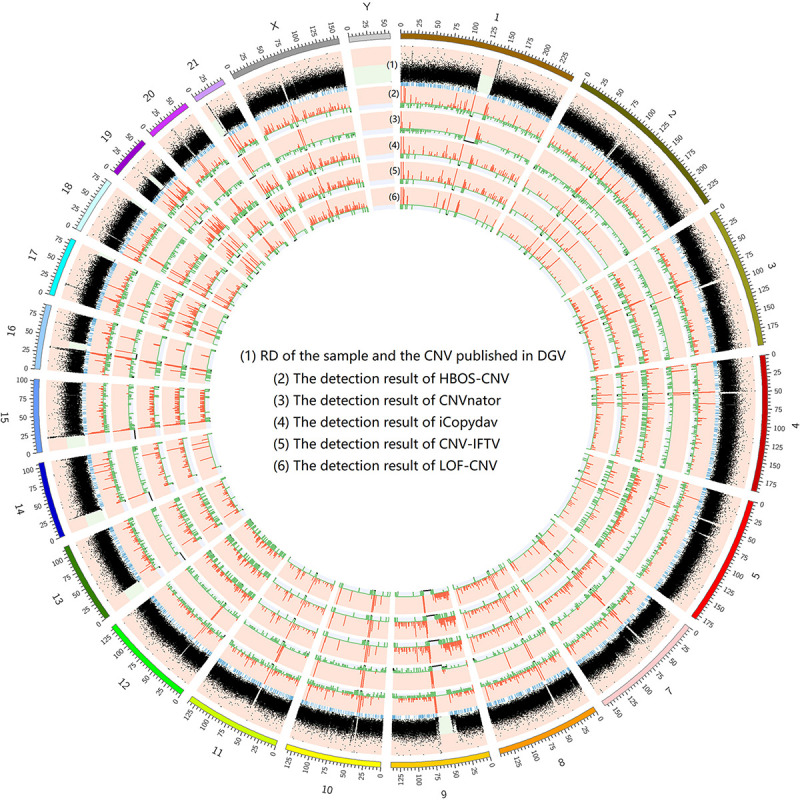
The detection of all whole genomes of NA19240 is shown, with the circles representing (1) RD, and CNV published in DGV (2) the CNVs detected with HBOS-CNV, (3) the CNVs detected with CNVnator, (4) the CNVs detected with iCopyDav, and (5) the CNVs detected with CNV-IFTV, (6) the CNVs detected with LOF-CNV, from the outside to the inside.

To show the CNV detection results more clearly, we use the circos map and a table to show the detection results of all samples. In Figures 7–9, scatter graphs show the RD information of the whole genome, blue lines represent the chromosome variation positions published in DGV. The CNV positions and absolute copy number of HBOS-CNV, CNVnator, iCopydav, CNV-IFTV, and LOF-CNV are displayed in the inner five cycles. Greenline represents the normal or lost region of CN, i.e., absolute copy numbers are less than or equal to 2. Redline represents the gain region of CN and black represents the position centromere telomere.

The results show that CNVnator has false positive detection in chromosome 1, chromosome 2, and chromosome X. Although the detection results of iCopydav have high precision, the total number of the variation is small and the sensitivity is low. HBOS-CNV and CNV-IFTV can detect most CNV in the three samples, and the results are relatively stable. But considering the running time, HBOS-CNV is much faster than CNV-IFTV.

The data in the Table 1 shows that the length of CNV detected by the five methods is different. The number of CNVs detected by CNVnator is greater than that of the other four methods and also greater than that reported by DGV. The detection precision and sensitivity of the other four methods are basically the same as those of the simulation data.

**TABLE 1:** Comparison of CNV detection quantity between HBOS-CNV and other methods on real samples.

Sample	HBOS-CNV	CNVnator	iCopydav	LOF-CNV	CNV-IFTV	DGV
NA19238	3882	6302	2153	3281	4552	3836
NA19239	4954	7442	2964	7435	5700	4842
NA19240	4434	7996	2930	4332	5407	4867

In summary, the HBOS-CNV method exhibited the optimal tradeoff among sensitivity, precision, and efficiency in this large real data application. Therefore, we concluded that HBOS-CNV is a reliable tool for the detection of CNVs.

## Discussion and Conclusion

The detection of CNVs can assist researchers in studying the origin and evolution of tumor genes from biological and medical perspectives, and can also be further used to isolate targeted drugs for the treatment of tumors (Li et al., 2009; Bellos and Coin, 2014; Boeva et al., 2014). To decrease the defects in the existing software, the current study presents a novel detection method HBOS-CNV, which can be used as a single tumor sample without a normal control-matched sample. Compared with the existing algorithms, HBOS-CNV possesses several new features, described as follows. (1) HBOS-CNV uses a sliding window to smooth the RD profile, which successfully reduces the interaction among the continuous bins, and increases the difference in RD values between normal regions and variation regions. (2) HBOS-CNV uses a dynamic width histogram to calculate the density of bins, which can reduce the influence of gain and loss amplitudes. For small-amplitude CNV regions, HBOS-CNV can calculate the density of local bins, and avoid the adverse effect of the maximum amplitude. (3) HBOS is a non-linearly transformed value obtained from the observed RD value of each genome bin, having the characteristics of fewer input variables and high stability for accurate calculation results.

Finally, the performance of HBOS-CNV was evaluated and verified by experiments. In the simulation experiment, the sensitivity, accuracy, and F1 measurement of HBOS-CNV were compared with the four existing methods. The detection results of CNVs with different purity and coverage depth were compared. The results showed that HBOS-CNV achieved the optimal trade-off among sensitivity, accuracy, and computational efficiency. In practical data application, HBOS-CNV was verified by results previously reported in the DGV database. The comparative results demonstrated that our approach has several advantages in terms of sensitivity, precision, F1-measure, and computational efficiency. Therefore, HBOS-CNV is expected to be a reliable tool to detect CNV from NGS data, especially for complex cases where the amplitude of CNV varies over a wide range.

The potential disadvantages of the HBOS-CNV method are discussed from the following two aspects. Firstly, HBOS-CNV uses a method that calculates the upper limit in a boxplot to determine the threshold of outliers and subsequently removes all bins smaller than the threshold. In some extreme gene sequencing data, this method may lead to a high false-positive rate. A more effective approach would be to preset the distribution model for the HBOS profile and calculate the significance of the data based on the model, so as to determine the outliers. Secondly, the GC bias correction method and the estimation method of tumor purity will limit the applicability of this algorithm. At present, the algorithm can only perform CNV detection on the sequencing data of human genes and does not apply to genes other than humans (Eberle et al., 2017; Turner et al., 2017). We plan to improve the algorithm in the next research.

In future work, we plan to solve the above problems to further improve the performance of HBOS-CNV, extend the method to the CNV detection of other animal and plant genes. This will be very helpful for studying the accurate quantification of CNV and exploring the evolution process of species (Zhu et al., 2017a; You et al., 2018). At the same time, we plan to use the CNV detection results in the correction of Cancer Cell Fraction (CCF), which will greatly promote a comprehensive understanding of tumor occurrence and development (Xi et al., 2018; Mao et al., 2021; Tarabichi et al., 2021). We will also try to apply this algorithm to the research of ancient DNA mutation detection, which may be helpful to explore the evolution process of species (Zhu et al., 2017b).

## Data Availability Statement

The original contributions presented in the study are included in the article/supplementary material, further inquiries can be directed to the corresponding author/s.

## Author Contributions

YG participated in the algorithm design and experiments, participated in the analysis of experimental results, and wrote the draft. YG and SW participated in the design of CNVs detection process. YG and XY conceived the research. XY guided the whole work. SW and XY helped to revise the draft. All authors read the final manuscript and agreed to submit it. All authors contributed to the article and approved the submitted version.

## Conflict of Interest

The authors declare that the research was conducted in the absence of any commercial or financial relationships that could be construed as a potential conflict of interest.

## Footnotes

1 http://www.internationalgenome.org/

2 http://dgv.tcag.ca/
